# CCL19: a novel prognostic chemokine modulates the tumor immune microenvironment and outcomes of cancers

**DOI:** 10.18632/aging.205184

**Published:** 2023-11-08

**Authors:** Qiang Gu, Shifang Zhou, Cong Chen, Zhi Wang, Wenhao Xu, Jiarong Zhang, Shiyin Wei, Jianfeng Yang, Hongjing Chen

**Affiliations:** 1 Affiliated Maternity and Child Health Care Hospital of Nantong University, Nantong 226000, China; 2 Department of Obstetrics and Gynecology, Zhongshan Hospital, Fudan University, Shanghai 200032, China; 3 Department of Nursing, Fudan University Shanghai Cancer Center, Shanghai 201321, China; 4 Department of Interventional Oncology, Renji Hospital, Shanghai Jiao Tong University School of Medicine, Shanghai 200120, China; 5 Department of Oncology, Shanghai Medical College, Fudan University, Shanghai 200032, China; 6 Affiliated Hospital of Youjiang Medical University for Nationalities, Baise 533000, China; 7 Department of Surgery, Shangnan Branch of Longhua Hospital Affiliated to Shanghai University of Traditional Chinese Medicine, Shanghai 200126, China

**Keywords:** cancer, CCL19, tumor microenvironment, CD8+ T cells, biomarker

## Abstract

Background: CCL19 is a chemokine involved in cancer research due to its important role in the tumor microenvironment (TME) and clinical relevance in cancers. This study aimed to analyze transcription expression, genomic alteration, association with tumor immune microenvironment of CCL19 expression and its prediction value for prognosis and responses to immunotherapy for patients with cancers.

Methods: RNA sequencing data and corresponding clinicopathological information of a total of large-scale cancer patients were obtained from The Cancer Genome Atlas and Gene Expression Omnibus databases. Multiplex immunofluorescence (mIF) was implemented to identify differential infiltration of Treg, CD8^+^ T cells, and tumor-associated macrophages, while CCL19 immunohistochemistry was conducted on 182 breast cancer samples from a real-world cohort.

Results: Based on large-scale multi-center survival analysis of cancer patients, we found the prognosis of patients with high CCL19 expression was prominently better than those with low CCL19 expression. For patients from multiple independent cohorts, suppressed CCL19 expression exerts significant progressive phenotype and apoptosis activity of cancers, especially in breast and ovarian cancer. Interestingly, anti-tumor immune cells, specifically the CD8^+^ T cells and macrophages, were clustered from TME by elevated CCL19 expression. Additionally, higher CCL19 levels reflected heightened immune activity and substantial heterogeneity.

Conclusions: In conclusion, our findings support the notion that elevated CCL19 expression is linked to favorable outcomes and enhanced anti-tumor immunity, characterized by increased CD8^+^ T cells within the TME. This suggests the potential of CCL19 as a prognostic marker, predictive biomarker for immunotherapy, therapeutic target of cancers.

## INTRODUCTION

Breast cancer (BRCA) is the most common cancer among women, accounting for approximately 2 million new cases annually, and is ranked as the fifth leading cause of cancer-related deaths worldwide [1–3]. Ovarian cancer (OV), particularly high-risk subtypes, is a formidable gynecological malignancy characterized by a high recurrence rate and mortality. Despite notable advancements in cancer therapeutics, these cancers continue to pose significant obstacles to effective treatment. Understanding these challenges is crucial for improving patient outcomes [4]. Traditionally, OV and BRCA have been managed through a combination of surgical interventions, radiotherapy, and chemotherapy. However, recent developments in the field have introduced immunotherapy as a promising addition to the treatment repertoire [5]. One of the primary challenges in treating BRCA and OV lies in the inherent genetic diversity among patients. Different driving genes govern the direction of cancer evolution at various stages, resulting in varying sensitivities to targeted therapies. This diversity of genetic factors has emerged as a major hurdle in achieving consistent success with cancer-targeted therapies [6]. Beyond genetic heterogeneity, tumors employ intricate mechanisms to evade the host immune system. These mechanisms can include the manipulation of immune checkpoints and the establishment of immunosuppressive microenvironments. Effectively countering these immune escape tactics is paramount for the success of immunotherapy approaches in cancers [7, 8].

Chemokines, a family of small molecules typically measuring about 8-10 kD in size, hold a vital role in orchestrating immune responses. These remarkable proteins act as molecular guides, attracting various cytokines, cells, and substances to specific locations within the body. Their influence extends across a wide array of biological processes, including the regulation of tumor microenvironment (TME) homeostasis, angiogenesis, tumor metastasis, immune responses, inflammation, and cell migration [9, 10]. Chemokines can be categorized into four distinct subfamilies based on the number of amino acids between the first two cysteine residues, and these subfamilies are CXC, CC, CX3C and XC. Within the realm of CC chemokines, chemokine (C-C motif) ligand 19 (CCL19) stands out. Positioned on the p-arm of chromosome 9, CCL19 is a leukocyte chemoattractant with significant roles to play. It facilitates the migration of T cells within the thymus and orchestrates the movement of both T cells and B cells towards secondary lymphoid organs [11]. Notably, CCL19 has been demonstrated to possess the remarkable ability to enhance CD8+ T cell responses. This means that it plays a crucial role in empowering the immune system to target and combat tumor cells effectively [12].

Recent research has delved into the intricate relationship between CCL19 expression and tumor prognosis, shedding light on its potential significance across various cancer types. Notably, prior studies have unearthed compelling evidence linking low CCL19 expression to unfavorable outcomes in cancers such as small cell lung cancer and follicular lymphoma. These findings underscore the pivotal role of CCL19 in shaping the clinical trajectory of cancer patients [13, 14]. Additionally, the intricate chemokine receptor 7 (CCR7) chemokine axis, characterized by chemokine ligand 21 (CCL21) and CCL19, has emerged as a promising avenue in cancer immunotherapy. This axis has demonstrated its capacity to be a potent target for therapeutic intervention [15, 16]. However, it’s important to note that CCR7 plays a dual role in cancer, sometimes promoting tumor progression and, at other times, bolstering antitumor immunity. This duality underscores the complexity of cancer therapy decision-making regarding the CCR7 axis [17].

In light of these insights, we venture to speculate that CCL19 expression levels may bear a significant relationship with the prognosis of cancer patients. We posit that CCL19 expression could exert control over immune cell dynamics, potentially influencing the efficacy of immunotherapy interventions. This, in turn, may usher in profound alterations in the prognostic landscape for affected patients.

## MATERIALS AND METHODS

### Data download and preprocessing from the public databases and Youjiang cohort

The RNA sequencing data of cancer patients were obtained from The Cancer Genome Atlas (TCGA, https://portal.gdc.cancer.gov) and Gene Expression Omnibus (GEO, https://www.ncbi.nlm.nih.gov/geo/) databases. The FPKM gene expression profile was measured experimentally using the Illumina HiSeq 2000 RNA Sequencing platform by the University of North Carolina TCGA genome characterization center. The clinical and pathological data of BRCA and OV patients were obtained from TCGA cohort. Protein expression of CCL19 in BRCA and OV were evaluated using the Human Protein Atlas (https://www.proteinatlas.org/). A total of 182 patients with breast cancer and collected medical records were included from Affiliated Hospital of Youjiang Medical University for Nationalities (Youjiang cohort) for further survival analysis.

### Measurement of association between CCL19 expression and clinicopathological indicators

Sanguini diagram was built based on the R software package “ggalluval”. The distribution of samples in higher and lower CCL19 expression groups was implemented by R foundation for statistical computing (2020) version 4.0.3 and “ggplot2” (v3.3.2) [18].

### Differential CCL19 expression and survival analysis of BRCA and OV

To assess the statistical significance of CCL19 mRNA expression between BRCA and adjacent normal samples, the Student’s t test was performed. The Kaplan-Meier method (95% confidence interval [95%CI]), log-rank test, univariate and multivariate Cox regression analyses were implemented to evaluate the significance of implications conferred upon prognosis of gene expression groups [19]. Besides, some survival plots were analyzed using the Kaplan-Meier Plotter (http://kmplot.com/analysis/index). The best performing threshold is computed and used as cutoff value according to online.

### Identifications and clustering of differential expressed genes with CCL19 expression of BRCA

Raw counts of level three RNA-seq data and corresponding clinicopathological information from 1097 BRCA samples were obtained from TCGA database, in which the method of acquisition and application complied with the guidelines and policies. “Adjusted p< 0.05 and Log (Fold Change) >1 or Log (Fold Change) < -1” were defined as the thresholds for the screening of differential expression of mRNAs [20]. The R software package “ConsensusClusterPlus” (v1.54.0) was used for consistency analysis, with the maximum number of clusters equal 6, and 80% of the total sample being drawn 100 times, clusterAlg = “hc”, innerLinkage=‘ward.D2’. The R software package “pheatmap” (v1.0.12) was used for clustering heatmap into two groups.

### Functional enrichment analysis

To further explore the underlying function of CCL19 in carcinogenesis and progression of BRCA, the differential expression genes were analyzed by functional enrichment. Gene Ontology (GO) is a widely-used tool for annotating genes with functions, especially molecular function (MF), biological pathways (BP), and cellular components (CC). Kyoto Encyclopedia of Genes and Genomes (KEGG) Enrichment Analysis is a practical resource for analytical study of gene functions and associated high-level genome functional information [21]. Then, “ClusterProfiler” package (version: 3.18.0) in R was employed to analyze the GO function of potential targets and enrich the KEGG pathway.

### Mutation abundance and frequency CCL19 expression in BRCA

We analyzed mutation abundance and frequency of CCL19 expression in BRCA using cBioportal for cancer genomics (http://www.cbioportal.org/) to identify significant associations between types of DNA copy number alterations of CCL19 and mRNA expression levels [22]. Genes expressed at significantly elevated levels in the CCL19 -altered and -unaltered groups were screened and identified using the Limma R package [23].

### Assessment of immune cells infiltration of BRCA and OV

CIBERSORT algorithm is an R/web version tool for deconvolution of the expression matrix of human immune cell subtypes based on the principle of linear support vector regression [24, 25]. The method is based on a known reference set that provides a set of gene expression signatures for 22 immune cell subtypes: LM22. In order to evaluate the reliability of the deconvolution method, “CIBERSORT” R package provides P value for each sample using default feature matrix with perm=100 times for analysis.

### Multispectral immunofluorescence (mIF) staining assays

Multispectral fluorescence immunohistochemistry (mIHC) staining was performed using the Akoya OPAL Polaris 7-Color Automation IHC kit (NEL871001KT) with two panels (Panel 1: CD8, CK, CD68, CD163, PD-1, PD-L1, DAPI; Panel 2: CD3, CK, CD56, CD20, CD4, FoxP3, and DAPI), achieved a total of 12 primary antibody staining imaging. CD163 (Abcam, ab182422, 1:500), CD68 (Abcam, ab213363, 1:1000), PD-1 (CST, D4W2J, 86163S, 1:200), PD-L1 (CST, E1L3N, 13684S, 1:400), CD3 (Dako, A0452), CD4 (Abcam, ab133616, 1:100), CD8 (Abcam, ab178089, 1:100), CD56 (Abcam, ab75813, 1:100), CD20 (Dako, L26, IR604), FoxP3 (Abcam, ab20034, 1:100) and pan-CK (Abcam, ab7753, 1:100) were obtained from Akoya Biosciences (Marlborough, MA, USA). Tissue slides that were bound with primary and secondary antibodies but not fluorophores were included as negative controls to assess autofluorescence. Multiplex stained slides were scanned using a Vectra Polaris Quantitative Pathology Imaging System (Akoya Biosciences) at 20 nm wavelength intervals from 440 nm to 780 nm with a fixed exposure time and an absolute magnification of 200X. All scans for each slide were then superimposed to obtain a single image.

### Immunohistochemistry (IHC) staining analyses

IHC staining was performed to assess the expression levels of CCL19 using primary antibodies against CCL19 (No. MA523833; Invitrogen, Thermo Fisher Scientific) and peroxidase-conjugated goat anti-rat IgG. Scoring of CCL19 expression in breast cancer tissues was implemented as previously described [26].

### Statistical analysis

Statistical analyses were performed using SPSS software (version 23.0, Inc, Chicago, IL, USA), GraphPad Prism 8.0, R software (version 3.4.3,), or online webtools. All the other methods and R package were implemented by R foundation for statistical computing (2020) version 4.0.3. All hypothetical analyses are two-sided, and *p* <0.05 indicates a significant difference.

### Consent for publication

Not applicable. The written informed consent was obtained from the online public database and AHYMUN committee.

### Availability of data and material

The datasets analyzed in this study were obtained from the corresponding author upon reasonable request or open-access online TCGA and GEO databases.

## RESULTS

### Assessing the prognostic significance of CCL19 expression in diverse cancer types

Our study aims to comprehensively evaluate the prognostic relevance of CCL19 expression in various cancer types. To achieve this objective, we initiated our investigation by examining the association between CCL19 expression levels and the overall survival (OS) (Supplementary Figure 1) and progression-free survival (PFS) (Supplementary Figure 2) among patients diagnosed with distinct cancer types.

We meticulously analyzed these trends within specific cancer categories to gain insights into the potential prognostic value of CCL19 expression. This systematic evaluation provides a foundation for elucidating the role of CCL19 as a prognostic marker in cancer and its implications for patient outcomes across different cancer types.

### Association of CCL19 expression with improved survival in breast and ovarian cancers

**Figure 1:**
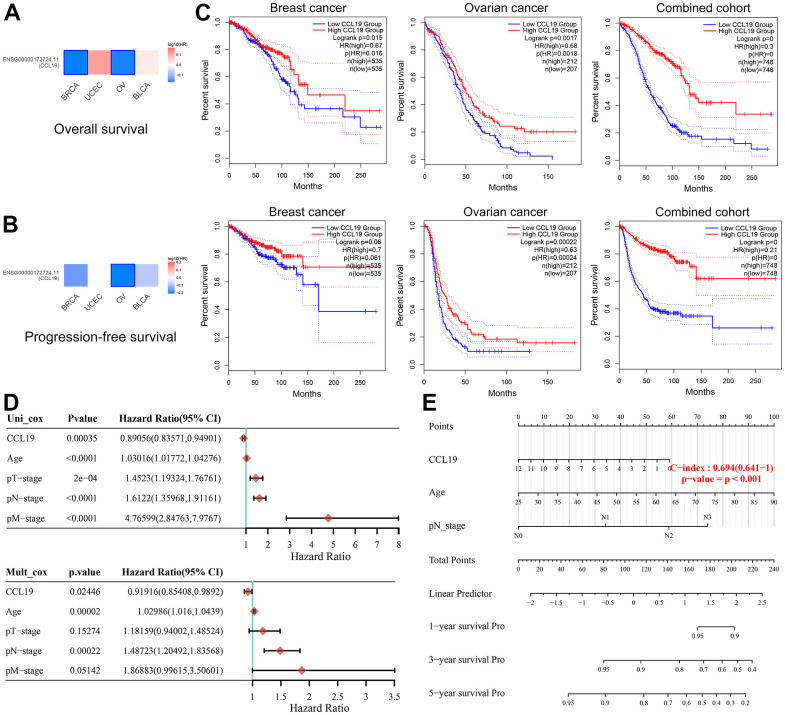
**Association of CCL19 expression with improved survival in breast and ovarian cancers.** (**A**, **B**) In BRCA and OV, the expression of CCL19 is associated with cancer suppression, manifested in better OS. Red represents a positive correlation with carcinogenesis, while blue represents cancer inhibition. (**C**) Kaplan-Meier survival analysis suggested the antitumor effect of CCL19 in patients with BRCA and OV (first row: PFS, second row: OS). (**D**) Univariate and multivariate Cox regression analysis indicated CCL19 expression as an independent prognostic biomarker for OS in patients with BRCA. (**E**) Robust nomogram was constructed to predict survival for patients with BRCA.

Our analysis, encompassing various cancer types sourced from the TCGA database, has unearthed a compelling relationship between CCL19 expression and cancer suppression, particularly evident in breast cancer (BRCA) and ovarian cancer (OV) cases (Figure 1A, 1B). Then, we embarked on an in-depth survival analysis of BRCA and OV cohorts, as well as a combined cohort. Among the 1070 BRCA samples, individuals exhibiting high CCL19 expression experienced significantly enhanced overall survival (OS) compared to their low CCL19 expression counterparts (p=0.016). Furthermore, while the difference was not pronounced, patients with high CCL19 expression also demonstrated improved progression-free survival (PFS) (p=0.061). In the case of OV, we observed a similar trend, with patients in the CCL19-high expression group exhibiting significantly superior OS (p=0.0018) and PFS (p=0.00024) compared to those with low CCL19 expression (Figure 1C).

In Figure 1D, univariate and multivariate Cox regression analysis indicated that CCL19 expression (p=0.024, HR=0.92) was an independent prognostic biomarker for OS in patients with BRCA, as well as age (p<0.001, HR=1.03) and the pathological N stage (p<0.001, HR=1.49). Robust nomogram was constructed to predicts survival for patients with BRCA (p<0.001, C-index=0.694) (Figure 1E).

When amalgamating all samples, the trend remains significant: elevated CCL19 expression is consistently associated with a more favorable prognosis for both BRCA and OV patients. These compelling findings underscore the potential role of CCL19 as a prognostic marker and its capacity to influence patient outcomes in BRCA and OV. However, it’s worth noting that precise statistical methods were employed to derive these results, reinforcing their validity and significance.

### Validation of prognostic implications of CCL19 in BRCA patients across independent cohorts

To ascertain the independent prognostic value of CCL19 expression, we conducted survival analyses using a robust dataset comprising a total of 1879 samples sourced from independent cohorts within the GEO database. These cohorts encompassed GSE1456, GSE2603, GSE6532, GSE11121, GSE12276, GSE19615, GSE48390, and GSE65194.

**Figure 2:**
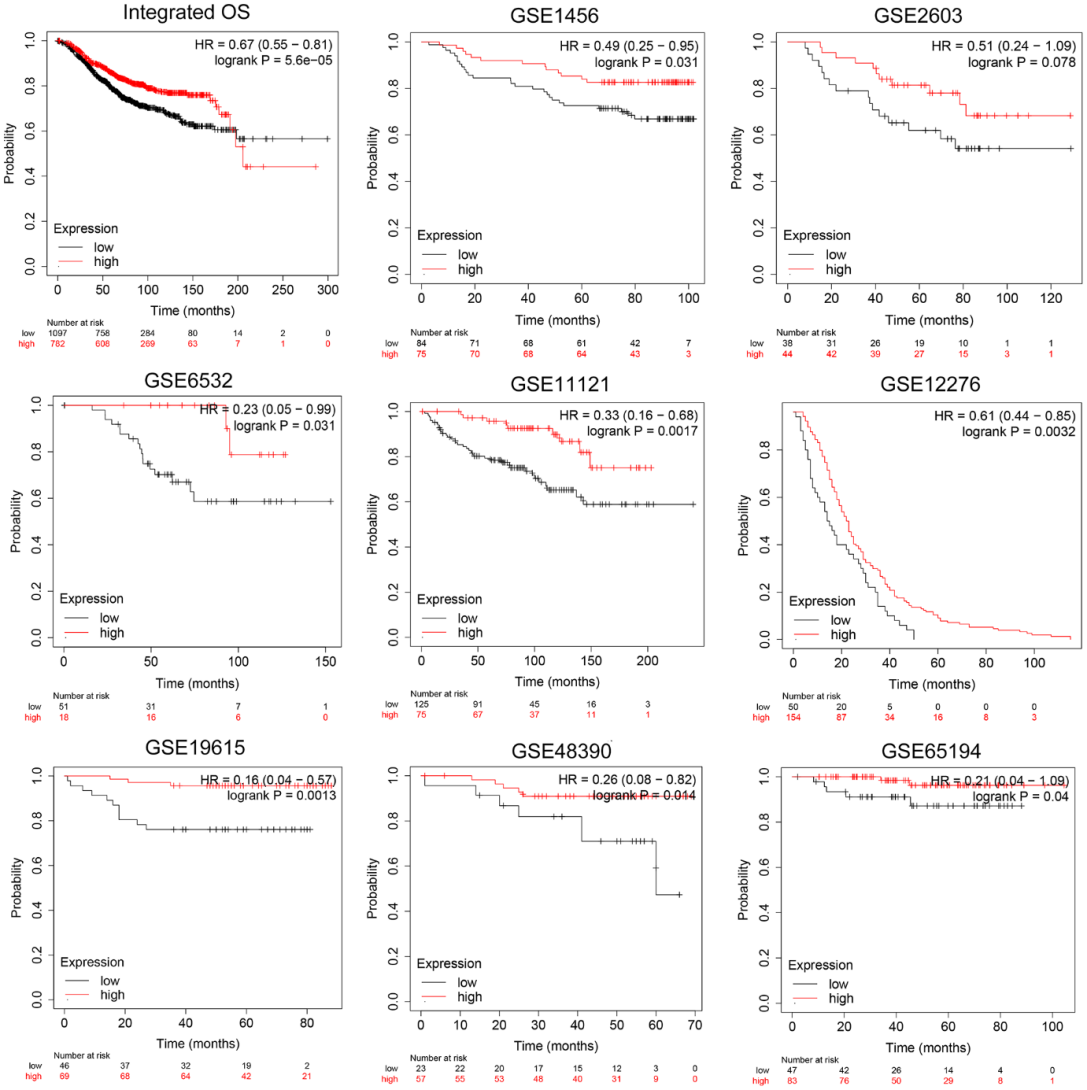
**Validation of prognostic implications of CCL19 expression for OS in BRCA patients across independent cohorts.** OS analysis of 1879 BRCA samples in total from independent cohorts in GEO database was performed.

Our analysis, as depicted in Figure 2, unequivocally demonstrates that patients exhibiting high CCL19 expression enjoy significantly prolonged overall survival (OS) when compared to their counterparts with lower CCL19 expression (p<0.05). This pattern holds true for each individual cohort, as well as when the data are integrated. These compelling findings consistently highlight CCL19 as a promising biomarker with the potential to predict the prognosis of BRCA patients. The statistical significance threshold of p<0.05 underscores the robustness of this association. These results collectively underscore the robust prognostic significance of CCL19 expression in BRCA patients, affirming its potential utility as a valuable biomarker for predicting patient outcomes across multiple independent cohorts.

**Figure 3:**
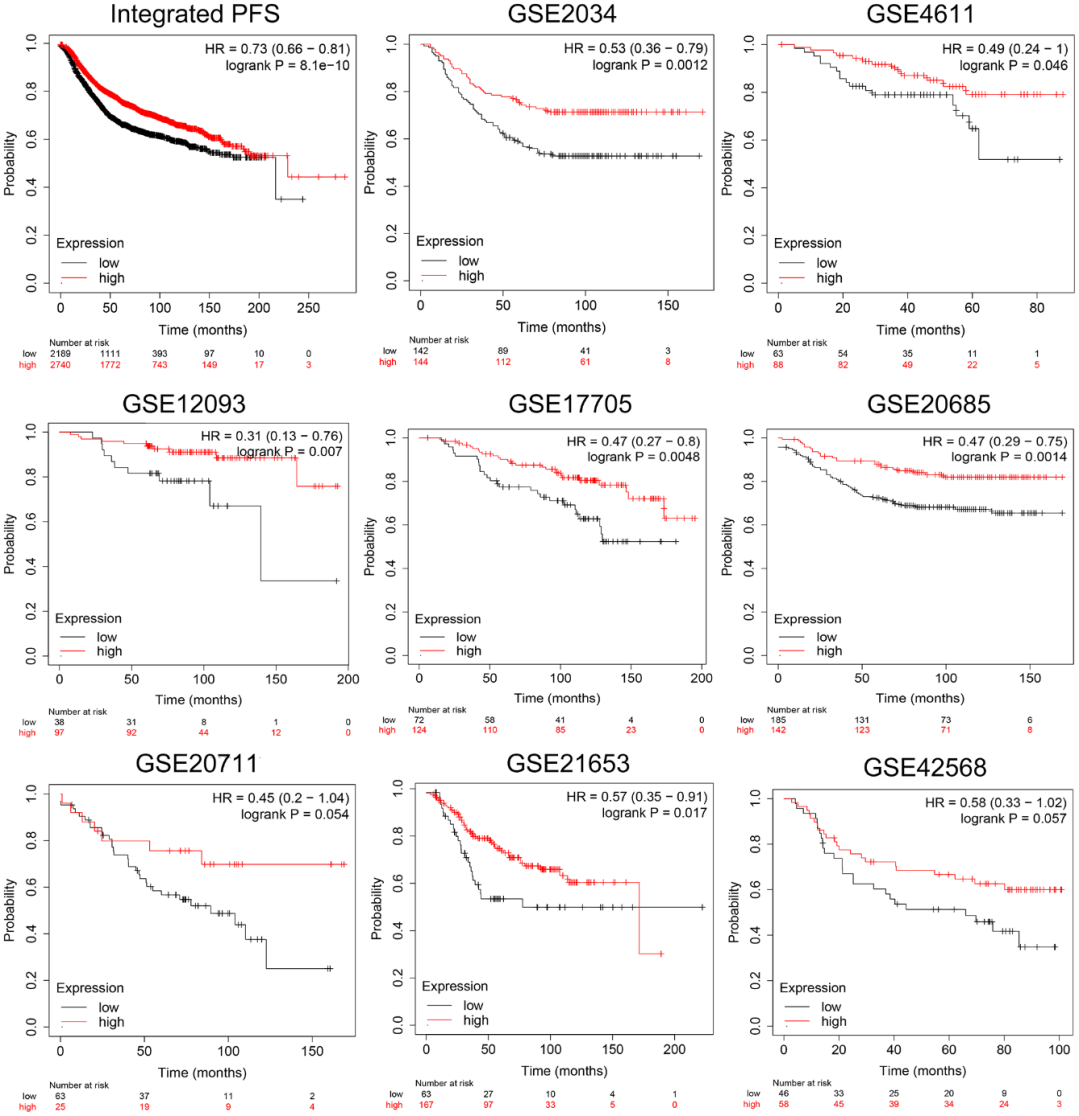
**Validation of prognostic implications of CCL19 expression for PFS in BRCA patients across independent cohorts.** With 4929 BRCA samples in total, PFS analysis of independent cohorts was performed.

To delve deeper into the connection between CCL19 expression and patient survival, we conducted a progression-free survival (PFS) analysis, thus eliminating the influence of non-disease-related factors. Our analysis encompassed a comprehensive dataset comprising a total of 4929 BRCA samples. The findings from independent cohorts consistently demonstrated improved PFS in patients with higher CCL19 expression levels. This observation held particular significance in cohorts such as GSE2034, GSE20685, and GSE17705 (p=0.0012; p=0.0014; p=0.0048; Figure 3). These compelling results robustly establish a correlation between elevated CCL19 expression and a favorable prognosis for BRCA patients. Having established this strong link between CCL19 expression and patient outcomes, further exploration of the clinical significance and biological implications of CCL19 is warranted to validate our conjectures.

### Suppressed CCL19 expression exert progressive phenotype and apoptosis activity of BRCA and OV

**Figure 4:**
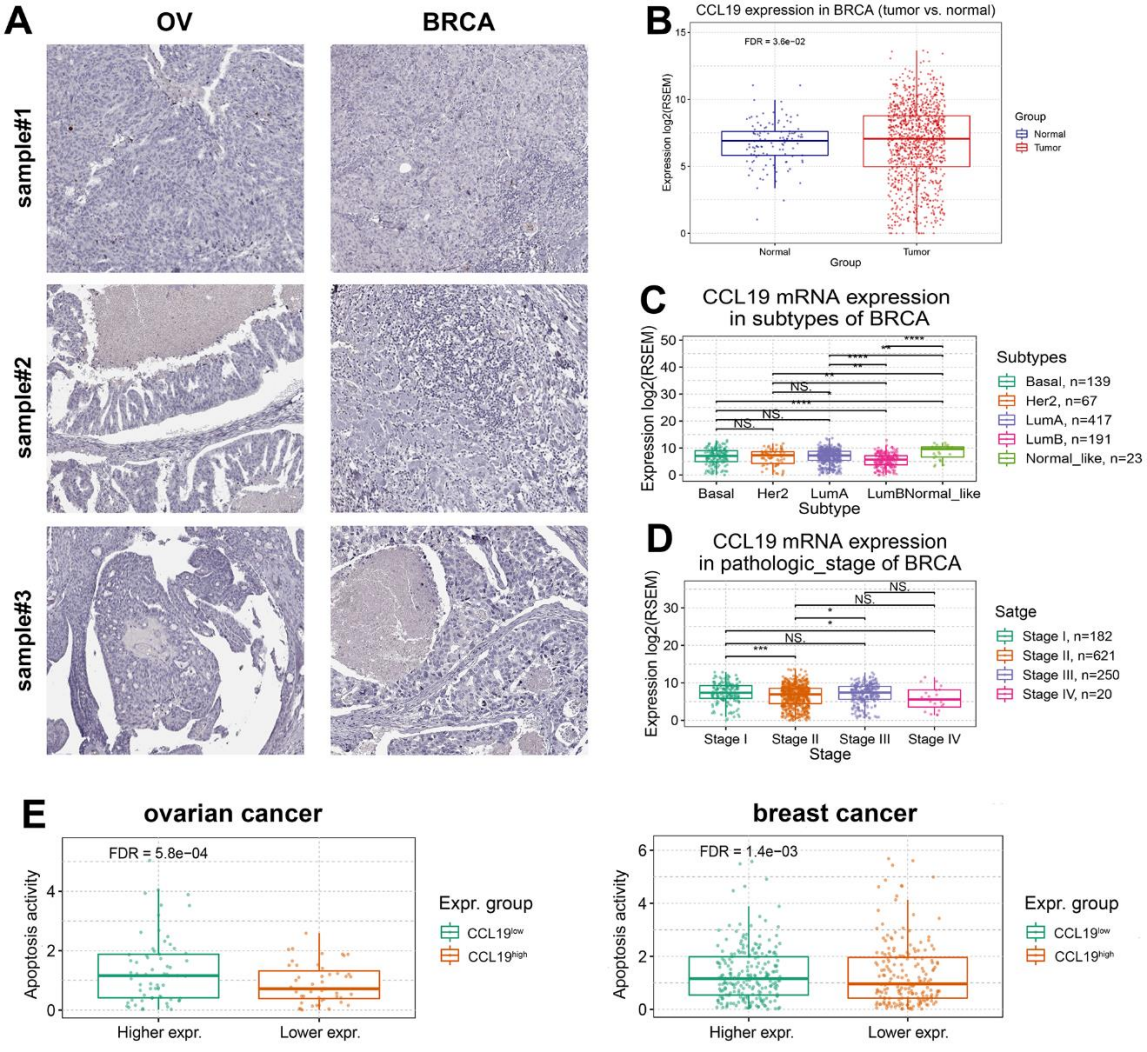
**Suppressed CCL19 expression exerts progressive phenotype and apoptosis activity of BRCA and OV.** (**A**) IHC analysis of BRCA and OV samples showed that the expression of CCL19 in normal tissues was higher than that in tumor tissues. (**B**) Analysis by software package RESM showed that the expression level of CCL19 in normal tissues is lower than that in tumor tissues in breast cancer samples. (**C**) The difference of CCL19 expression in different subtypes of breast cancer was analyzed. (**D**) The expression of CCL19 in four clinical stages was observed and compared, and the expression of CCL19 in stage 4 patients was the lowest. (**E**) Apoptosis activity was tested in CCL19^high^ group and CCL19^low^ group in BRCA and OV. With higher expression of CCL19, the apoptosis activity is higher.

Subsequently, we explored the clinical value of CCL19 expression of BRCA and OV. Histological observation showed that the content of CCL19 in normal tissues was significantly higher than that in tumor tissues, indicating that short of CCL19 may contribute to the occurrence of BRCA and OV (Figure 4A). Our initial histological observations revealed intriguing disparities in CCL19 expression between normal and tumor tissues. Notably, in OV, the content of CCL19 in normal tissues was significantly higher than that in tumor tissues, hinting at a potential role for reduced CCL19 in the development of OV (Figure 4A). However, the situation in BRCA samples was distinct, with CCL19 expression levels in normal tissues being lower than those in tumor tissues (Figure 4B).

Further analysis encompassed the examination of various BRCA subtypes, including Basal, Her2, LumA, LumB, and Normal_like, involving a total of 837 samples. Interestingly, we observed considerable variations in CCL19 expression levels among these subtypes (Figure 4C). We further investigated the relationship between CCL19 expression and the pathological stages of BRCA. Patients were categorized into four stages, ranging from stage 1 (least severe) to stage 4 (most severe). Intriguingly, CCL19 expression levels exhibited stage-dependent variation, with the lowest expression observed in stage 4, suggesting a potential negative correlation between CCL19 expression and cancer severity (Figure 4D). A noteworthy finding emerged when examining apoptosis activity in relation to CCL19 expression. Higher CCL19 expression was associated with markedly elevated apoptosis activity. This phenomenon is primarily attributed to CCL19’s ability to activate immune cells, such as CD8+ T cells, which subsequently engage cancer cells, leading to apoptosis (Figure 4E). Through our comprehensive analysis and research, a compelling association between high CCL19 expression and improved clinical prognosis begins to emerge. These findings underscore the potential clinical significance of CCL19 as a prognostic marker in BRCA and OV.

### Correlation between CCL19 expression and anti-tumor immune cells

**Figure 5:**
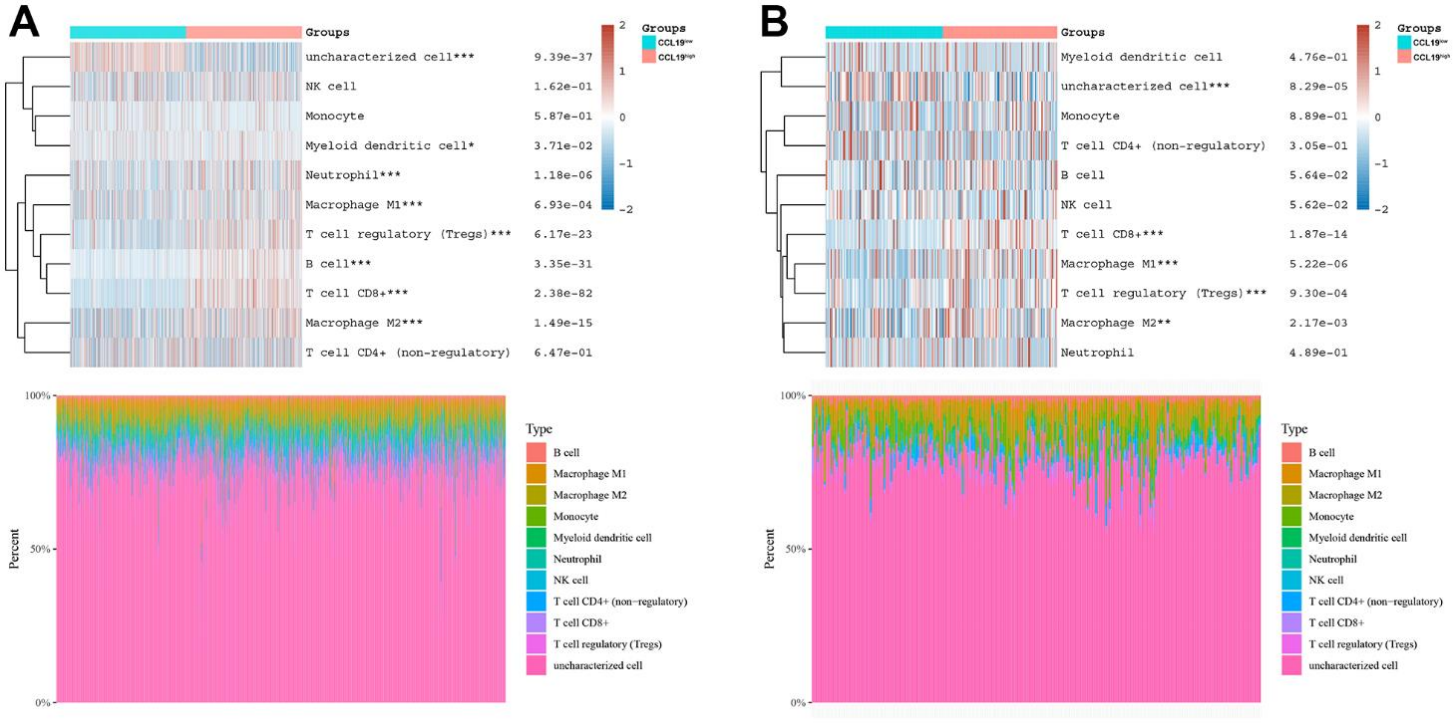
**Correlation between CCL19 expression and anti-tumor immune cells.** (**A**) BRCA patients were clarified into two groups, CCL19^high^ group and CCL19^low^ group. Then the correlation between immune cells and CCL19 expression was studied. Each line is a sample. Red indicates positive correlation, the corresponding immune cells are easy to be activated, while blue indicates that the high/low expression of CCL19 has a negative impact on immune cells. (**B**) We classified OV patients, and then studied the effects of CCL19 high expression environment and low expression environment on different immune cells.

Having explored the prognostic implications of CCL19 expression, our focus shifted to understanding its relationship with immune cell activity in breast cancer (BRCA) and ovarian cancer (OV). In our analysis of BRCA samples, patients were categorized into CCL19^high^ and CCL19^low^ groups. This division allowed us to examine immune cell activity in these distinct groups. Notably, the CCL19^high^ group exhibited heightened activity among immune cells, particularly in Macrophage M1 cells, B cells, and CD8^+^ T cells, compared to the CCL19^low^ group (Figure 5A). Similarly, in the context of OV samples, we observed a significant difference in the activity of CD8^+^ T cells between the CCL19^high^ and CCL19^low^ groups. This observation indicates that the presence of high CCL19 expression might enhance the effectiveness of immunotherapy, particularly with regard to CD8^+^ T cell activity (Figure 5B).

Interestingly, single-cell RNA-seq data revealed expression location of CCL19 in the TME of breast cancer. After identification of 2,472 cells from 8 BRCA patients using single-cell SRP114962 cohort, we found that CCL19 was mainly expressed in CD8+ T cells, and CD8+ T cell highly cover the tumor (Supplementary Figure 3).

### Immunological response and substantial heterogeneity occurred within BRCA and OV in accordance with CCL19 expression

**Figure 6:**
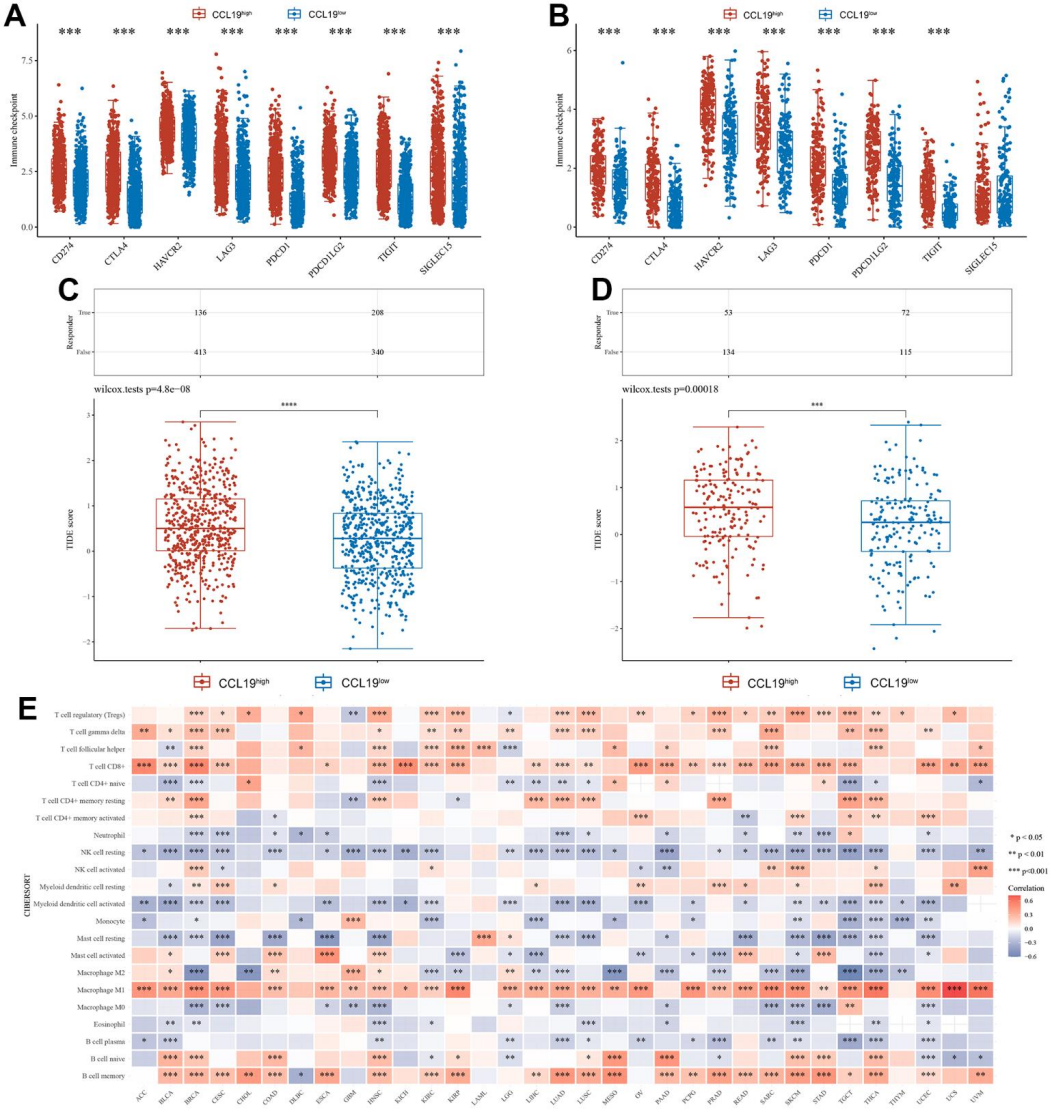
**Immunological response and substantial heterogeneity occurred within BRCA and OV in accordance with CCL19 expression.** (**A**) We studied several checkpoints expression level under high CCL19 expression and low expression in BRCA. (**B**) The difference of immune checkpoint between high expression group and low expression group of CCL19 in OV samples was also compared. (**C**) Tumor immune dysfunction and exclusion (TIDE) test was performed to check the immune infiltration in high expression group and low expression group of CCL19 in BRCA samples. (**D**) The difference of immune infiltration between high expression group and low expression group of CCL19 in OV was found with TIDE test. (**E**) We studied the correlation between CCL19 expression level and different immune cells in pan cancer. Red indicates a positive correlation between the expression of CCL19 and the activity of corresponding immune cells, while blue indicates a negative correlation.

Our investigation delved deeper into the influence of CCL19 expression on immune activities, shedding light on its significance in breast cancer (BRCA), ovarian cancer (OV), and across various cancer types. To assess the effects of CCL19 expression on immune responses, we examined the expression levels of common immune checkpoints in varying CCL19 environments. In both BRCA and OV samples, the CCL19^high^ group exhibited notably higher expression levels of immune checkpoints compared to the CCL19low group. This difference was particularly remarkable for PDCD1 and TIGIT in BRCA and HAVCR2, LAG3, and PDCD1LG2 in OV (Figure 6A, 6B). Furthermore, we conducted a Tumor Immune Dysfunction and Exclusion (TIDE) test to evaluate immune infiltration. The CCL19 high-expression group displayed higher TIDE scores, indicative of stronger tumor immune function. These findings suggest that CCL19 could serve as a potential biomarker or regulator of immune checkpoint inhibition resistance in BRCA and OV (Figure 6C, 6D).

In a broader pan-cancer context, we explored the correlation between CCL19 expression levels and various immune cell types. CD8^+^ T cells, Macrophage M1 cells, and B cell memory demonstrated significant positive correlations with most cancers. These immune cell types appeared to be activated and functional across a range of cancers, extending beyond BRCA and OV. Conversely, NK cells at rest and Macrophage M0 showed negative correlations with most cancers (Figure 6E). This implies that CCL19-activated tumor microenvironments might inhibit the activity of these immune-effective cells in cancers. These findings underscore the multifaceted role of CCL19 in shaping the immune landscape within tumor microenvironments and have potential implications for cancer immunotherapy and future research endeavors.

### CCL19 modulates tumor immune microenvironment features and predicts prognosis in breast cancer patients

**Figure 7:**
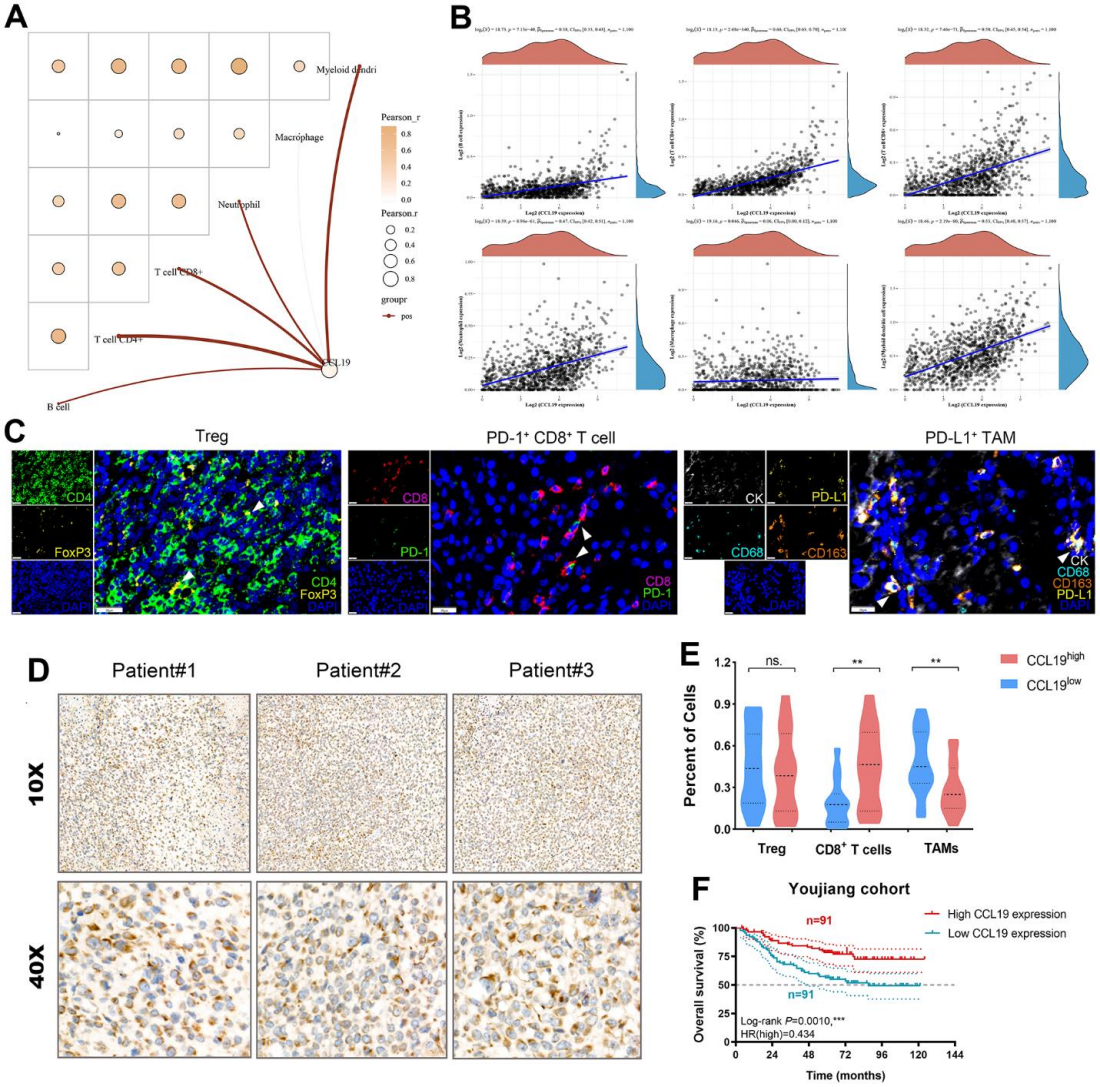
**CCL19 modulates tumor immune microenvironment features and predicts prognosis in breast cancer patients.** (**A**) We implemented with the CIBERSORT algorithms and identified prominently positive association between CCL19 expression and tumor-associated immune cells, including B cells, CD4+ T cells, CD8+ T cells, neutrophil, and myeloid dendritic cells. (**B**) Then, Sperman’s correlations between CCL19 expression and the immune cells infiltration were shown in the form of dot plots. (**C**) Multiplex fluorescence was implemented to identify Treg, CD8^+^ T cells, and PD-L1^+^ macrophages. (**D**) IHC staining of CCL19 in a total of 182 patients with breast cancer. (**E**) Differential percent of specific cells were compared between CCL19 expression groups using Student’s t test. (**F**) Kaplan-Meier survival analysis revealed that higher expression of CCL19 significantly predicts long-term outcomes for breast cancer patients.

Next, to reveal the association between CCL19 expression and the immune cells infiltration levels, we implemented with the CIBERSORT algorithms and identified prominently positive association between CCL19 expression and tumor-associated immune cells, including B cells, CD4+ T cells, CD8+ T cells, neutrophil, and myeloid dendritic cells (Figure 7A). Then, Sperman’s correlations between CCL19 expression and the immune cells infiltration were shown in the form of dot plots (Figure 7B).

Our investigation extended to a cohort of 182 breast cancer patients from the Affiliated Hospital of Youjiang Medical University for Nationalities (Youjiang cohort). In this segment, we sought to evaluate the relationship between CCL19 expression, the tumor immune microenvironment, and patient prognosis. We began by quantitatively assessing various lymphocyte populations for tumoral and peritumoral infiltration. This included regulatory T cells (Tregs), PD-1^+^ CD8^+^ T cells, and PD-L1^+^ tumor-associated M2 macrophages (PD-L1^+^ TAMs) (Figure 7C). Immunohistochemistry (IHC) staining demonstrated elevated CCL19 protein expression in breast cancer tissues (Figure 7D). Our analysis revealed intriguing insights. While there was no statistically significant difference in Treg infiltration between patient groups with high or low CCL19 expression, we observed a significantly higher abundance of CD8^+^ T cells and lower infiltration of TAMs in the CCL19^high^ group (Figure 7E). Kaplan-Meier survival analysis yielded compelling results. Higher expression of CCL19 emerged as a significant predictor of favorable long-term outcomes for breast cancer patients (HR=0.434, p=0.001; Figure 7F).

These findings underscore the complex interplay between CCL19 expression, the immune microenvironment, and breast cancer prognosis. Further exploration of these relationships may hold promise for refining treatment strategies and improving patient outcomes.

## DISCUSSION

The current landscape of surgical treatment for breast cancer (BRCA) and ovarian cancer (OV) is still evolving, with systemic treatment playing a crucial role as an adjunctive approach [3]. However, despite advancements in treatment modalities, patients continue to face challenges, including poor prognosis and disease progression. Immunotherapy has shown promise in addressing these challenges, yet it remains hindered by the absence of robust biomarkers and the significant individual variations among patients. Efforts have been made to identify biomarkers that can guide immunotherapeutic strategies. Among these efforts, several members of the CC chemokine ligand (CCL) family have shown promise. These molecules have emerged as key factors in vascular and tissue injury associated with chronic respiratory diseases. Notably, studies have revealed a significant increase in CCL18 and CX3CL1 levels in patients with conditions such as chronic obstructive pulmonary disease (COPD) and chronic cough with phlegm (CCP) when compared to healthy individuals [27]. Research has explored the therapeutic potential of CCL21 in the context of breast cancer (BRCA) [28], suggesting its significance in cancer treatment. Dysregulation of CCL19 has been noted in several cancers, including colorectal, pancreatic, and lung cancers, where it has been considered a potential tumor biomarker for diagnosis and prognosis [29]. These findings underscore the importance of investigating CCL family genes as potential biomarkers for tumor immunotherapy. The identification of reliable biomarkers holds the promise of improving treatment precision and patient outcomes in the challenging landscape of BRCA and OV management.

Despite the prevalence of breast cancer (BRCA) and ovarian cancer (OV), there has been a conspicuous gap in our understanding of the prognostic implications of CCL19 in these malignancies. Our groundbreaking research marks the first of its kind, revealing CCL19 as a promising tumor suppressor gene with profound prognostic value in BRCA and OV. We embarked on this scientific journey with the commitment to establish the clinical relevance of CCL19 markers, drawing upon an extensive array of external cohorts for rigorous validation. Our study harnessed the power of complex machine learning algorithms to delve into the intricate immune microenvironment orchestrated by CCL19. Notably, our findings illuminate a compelling connection between heightened CCL19 expression and the recruitment of M1 macrophages and CD8+ T cells. This recruitment signifies a potent antitumor immune response within samples boasting elevated CCL19 expression. Furthermore, our investigation aligns seamlessly with prior studies focusing on CD8+ T cells. These studies have unraveled critical insights into T cell clonality, T cell subset distribution, and antigen presentation within distinct BRCA subtypes. The implications of these findings are profound, offering a rationale for subtype-specific combination immune therapies [30, 31]. In summary, our research underscores the pivotal role of CCL19 in shaping the immune microenvironment. Environments characterized by robust CCL19 expression demonstrate the remarkable ability to mobilize immune cell reinforcements, notably CD8+ T cells and M1 macrophages, in the relentless battle against tumors. This substantiates the potential of CCL19 as a prognostic indicator and a guiding star in the realm of immunotherapy.

CCL19, a pivotal chemokine, plays a critical role in orchestrating the migration of immune cells toward lymphoid tissues. In the context of tumors, CCL19 often emerges as a key player, emanating not only from tumor cells but also from other constituents of the tumor microenvironment. Its functional significance lies in its binding to C-C chemokine receptor 7 (CCR7), a receptor that serves as a beacon guiding immune cells into the heart of the tumor tissue. The realm of breast cancer has been a focal point in the exploration of the CCL19/CCR7 pathway. For example, previous study described a 12-chemokine gene signature, and identified CCL19 as key chemokine for the maturation heterogeneity of tertiary lymphoid structures (TLS) in renal cell carcinoma, reflecting different TME immunological status and prognosis of cancers [10]. Extensive research has unveiled a multifaceted role for CCL19 within this context, with far-reaching implications for tumor progression. In the intricate dance of breast cancer, CCL19 has been implicated in a spectrum of activities, ranging from the potentiation of tumor cell growth to the facilitation of invasion and metastasis [32]. Specifically, the CCL19/CCR7 signaling pathway assumes a multifunctional role in breast cancer. It exerts its influence by promoting the proliferation and survival of tumor cells, all while enhancing their invasive and migratory prowess. Moreover, this signaling axis stirs the activity of immune cells within the tumor microenvironment, a cast that includes dendritic cells and T lymphocytes. In doing so, it further amplifies the invasive and metastatic potential of breast cancer cells [31].

The CCL19/CCR7 pathway emerges as a pivotal player in breast cancer, notably in the context of lymph node metastasis, and orchestrates a series of intricate molecular mechanisms [33]. This pathway hinges on the binding of CCL19 to its receptor, CCR7, expressed on the surfaces of breast cancer cells [34]. This interaction serves as the ignition point for a cascade of downstream signaling pathways, most notably the mitogen-activated protein kinase (MAPK) and phosphoinositide 3-kinase (PI3K)/Akt pathways [35].

The activation of these pathways carries profound implications for tumor progression. The MAPK pathway activation, for instance, sets in motion a sequence of events, including the activation of transcription factors such as activator protein-1 (AP-1) and nuclear factor-kappa B (NF-κB). These factors, in turn, drive cell proliferation and bolster cell survival. Simultaneously, the PI3K/Akt pathway activation comes into play, further fortifying cell survival mechanisms while curbing apoptosis. These combined effects contribute to the relentless growth of breast cancer cells. Beyond these pivotal pathways, the CCL19/CCR7 signaling axis exerts its influence by inducing the expression of matrix metalloproteinases (MMPs), enzymes with a knack for degrading the extracellular matrix. This degradation process paves the way for enhanced cell migration and invasion, facilitating the spread of breast cancer cells [36]. Adding to this complex interplay, the upregulation of integrins—cell adhesion molecules—promotes cell migration, adding to the dynamic of tumor progression. Additionally, the CCL19/CCR7 signaling cascade triggers the secretion of cytokines and chemokines, including interleukin-6 (IL-6) and vascular endothelial growth factor (VEGF). These secreted factors further amplify the proliferation and migration of tumor cells, stimulate angiogenesis, and wield their influence over the ever-evolving tumor microenvironment in breast cancer [37].

While our study has shed light on the significant role of CCL19 in breast cancer, we acknowledge certain limitations. Specifically, the absence of molecular experiments to validate the biological significance of CCL19 in cancer cell malignancy is notable. To overcome this limitation, we undertook an extensive approach, leveraging large sample sizes and pan-cancer data. These resources allowed us to harness transcriptome information to predict the intricate characteristics of CCL19’s involvement in the immune microenvironment. Through the application of complex machine learning algorithms, we delved into the potential functions of CCL19 in breast cancer (BRCA), ovarian cancer (OV), and pan-cancer contexts. Our findings underscore the pivotal role of CCL19 in driving the malignant progression of breast cancer. CCL19 emerges as a promoter of tumor cell growth, invasion, and metastasis, alongside its remarkable ability to stimulate immune cells within the tumor microenvironment. The molecular mechanisms orchestrating these effects primarily revolve around the activation of the CCL19/CCR7 signaling pathway. This activation, in turn, triggers a cascade of downstream signaling pathways, fosters the expression of matrix metalloproteinases (MMPs) and integrins, and fuels the secretion of cytokines and chemokines that bolster tumor cell proliferation and migration.

Looking ahead, these insights hold significant promise for the prevention and treatment of breast cancer. While we recognize the need for further research to unlock the full spectrum of this pathway’s potential, we anticipate that it will unveil new strategies and therapeutic targets that could revolutionize the management of breast cancer. The journey towards a deeper understanding of CCL19 in breast cancer continues, paving the way for innovative approaches to combat this formidable disease.

## CONCLUSIONS

In conclusion, our findings support the notion that elevated CCL19 expression is linked to favorable outcomes and enhanced anti-tumor immunity, characterized by increased CD8^+^ T cell and M1 macrophage presence. CCL19 plays a significant role in cancer research by influencing immune cell recruitment, activation, and the formation of tertiary lymphoid structures within the TME. This suggests the potential of CCL19 as a prognostic marker and immunotherapy target in breast and ovarian cancers. Further research into the complex interactions involving CCL19 in different cancer types will likely yield valuable insights for cancer diagnosis, prognosis, and treatment strategies.

## Supplementary Material

Supplementary Figures
