# Supplementary material for: CCL19: a novel prognostic chemokine modulates the tumor immune microenvironment and outcomes of cancers
Source: Aging (Albany NY). 2023 Nov 8;15(21):12369–87. doi: 10.18632/aging.205184 (PMC10683612; doi:10.18632/aging.205184)
Supplement: Supplementary Figures [file aging-15-205184-s001.pdf]

SUPPLEMENTARY FIGURES

Cancer	Pvalue	Hazard Ratio(95% CI)
ACC	0.3358	1.4418(0.68439,3.03742)
BLCA	0.7728	1.04449(0.77732,1.40349)
BRCA	0.0137	0.66938(0.48654,0.92092)
CESC	0.0790	0.65781(0.41223,1.04968)
CHOL	0.2785	0.58079(0.21738,1.5517)
COAD	0.5935	0.89819(0.60556,1.33223)
DLBC	0.8374	1.16336(0.2743,4.93401)
ESCA	0.1703	0.70627(0.42965,1.16099)
GBM	0.3094	0.83062(0.58077,1.18795)
HNSC	0.1968	0.8385(0.64171,1.09563)
KICH	0.7888	0.83533(0.2239,3.11641)
KIRC	0.4752	1.11456(0.8276,1.50102)
KIRP	0.0254	2.06218(1.09304,3.89059)
LAML	0.2250	0.76823(0.50178,1.17618)
LGG	0.7509	0.94443(0.66354,1.34422)
LIHC	0.6175	0.91564(0.64789,1.29402)
LUAD	0.3037	0.85869(0.64233,1.14794)
LUSC	0.2942	1.15568(0.88193,1.51441)
MESO	0.5207	0.85827(0.53838,1.36823)
OV	0.0156	0.7253(0.55901,0.94105)
PAAD	0.3419	0.81934(0.54324,1.23576)
PCPG	0.4360	0.50936(0.09328,2.78126)
PRAD	0.8203	1.16117(0.31988,4.21504)
READ	0.1505	0.55509(0.24882,1.23836)
SARC	0.0055	0.56459(0.37708,0.84536)
SKCM	0.0556	0.76688(0.58438,1.00637)
STAD	0.3870	1.1557(0.83264,1.60411)
TGCT	0.2027	4.49142(0.44532,45.29931)
THCA	0.5839	0.75868(0.28237,2.03847)
THYM	0.1428	3.24628(0.67214,15.67881)
UCEC	0.3633	1.21115(0.80139,1.83045)
UCS	0.0988	1.8122(0.89456,3.67118)
UVM	0.6066	1.24324(0.54277,2.8477)

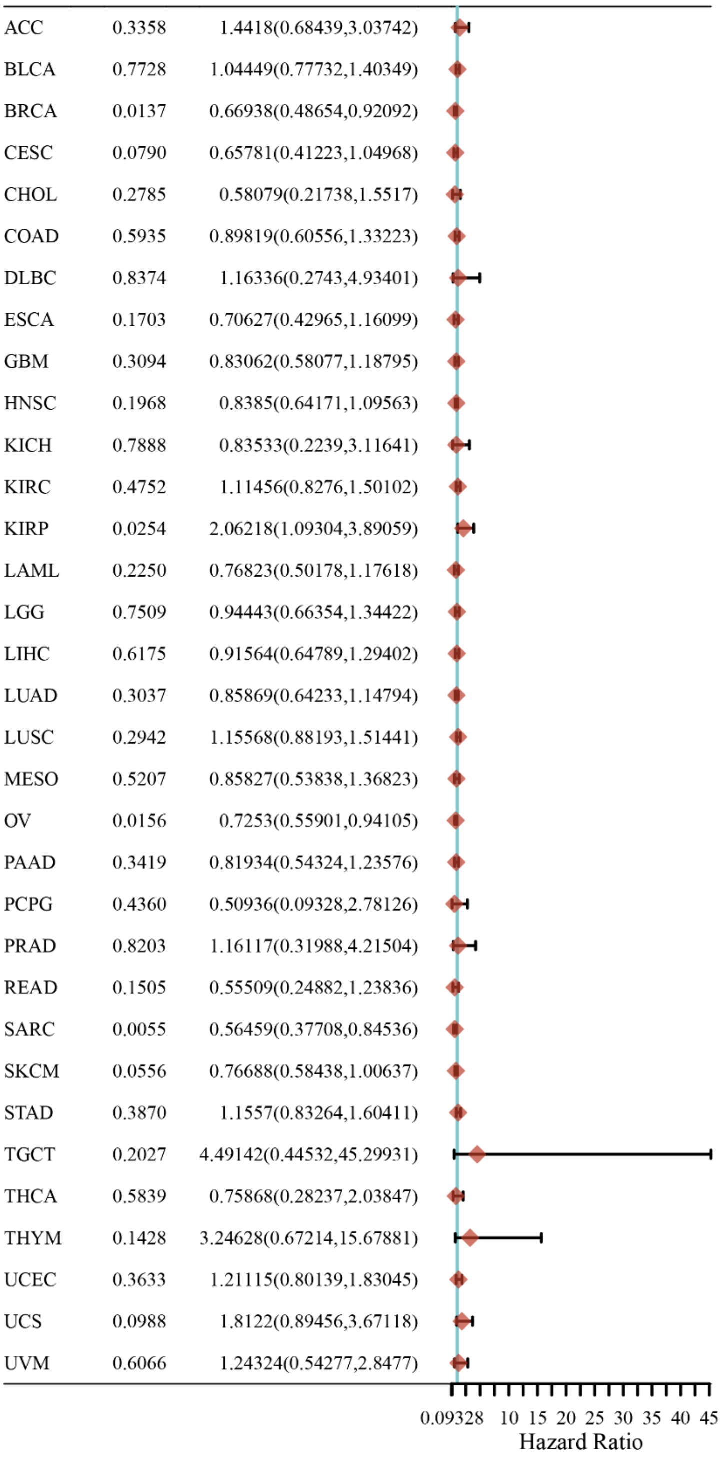

Supplementary Figure 1. Univariate Cox regression analysis revealed clinical value of expression level of CCL19 in predicting OS for patients with cancers in a forest plot.

Cancer	Pvalue	Hazard Ratio(95% CI)
ACC	0.7377	1.11053(0.6012,2.05136)
BLCA	0.2441	0.83737(0.62117,1.12882)
BRCA	0.0147	0.66646(0.48106,0.92332)
CESC	0.0365	0.60463(0.3773,0.96893)
CHOL	0.0195	0.3076(0.11436,0.82736)
COAD	0.1290	0.75688(0.52827,1.08442)
DLBC	0.4546	0.63329(0.19122,2.09732)
ESCA	0.9029	0.97298(0.62673,1.51054)
GBM	0.4945	0.88241(0.61633,1.26337)
HNSC	0.1375	0.8063(0.60686,1.07128)
KICH	0.7676	0.83576(0.25427,2.74701)
KIRC	0.5347	1.10383(0.80807,1.50783)
KIRP	0.0098	2.09737(1.19566,3.67909)
LGG	0.1537	0.81285(0.61148,1.08054)
LIHC	0.0912	0.77648(0.57894,1.04144)
LUAD	0.0860	0.78628(0.59755,1.03462)
LUSC	0.8768	0.97458(0.70388,1.34939)
MESO	0.0736	0.62247(0.3703,1.04637)
OV	0.0024	0.6898(0.54257,0.87698)
PAAD	0.8389	0.96073(0.65291,1.41368)
PCPG	0.9291	1.03991(0.43902,2.46324)
PRAD	0.5789	1.12188(0.74744,1.6839)
READ	0.8017	0.91956(0.47784,1.76961)
SARC	0.0655	0.73066(0.52323,1.02032)
SKCM	0.2288	0.87092(0.69537,1.09077)
STAD	0.0454	1.43869(1.00741,2.05461)
TGCT	0.8423	0.93443(0.47909,1.82251)
THCA	0.9748	0.99144(0.58148,1.69046)
THYM	0.0461	2.62302(1.01704,6.76491)
UCEC	0.2564	1.22702(0.8618,1.747)
UCS	0.2594	1.46359(0.75497,2.83731)
UVM	0.2358	0.63653(0.30164,1.34323)

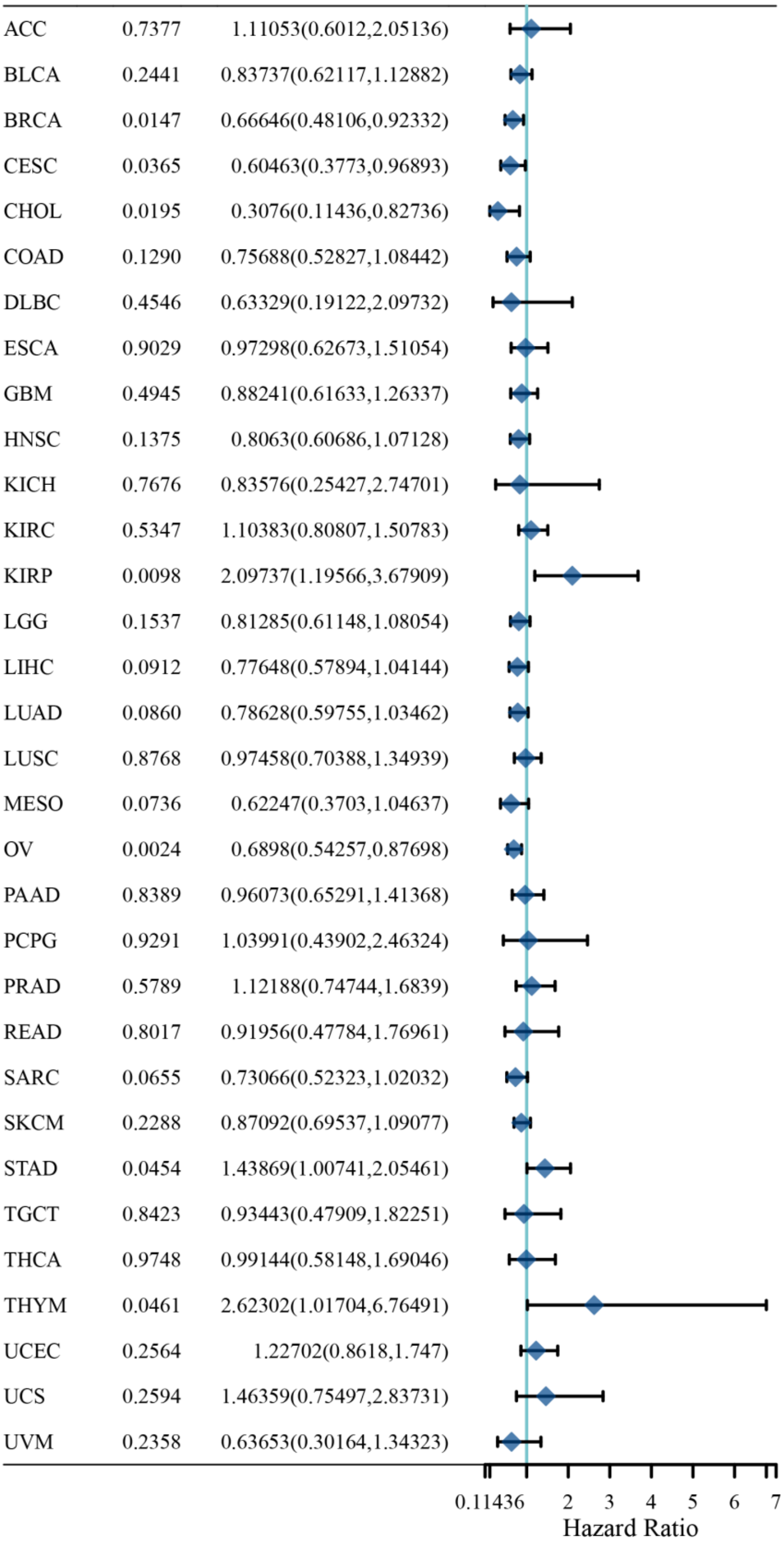

Supplementary Figure 2. Univariate Cox regression analysis revealed clinical value of expression level of CCL19 in predicting PFS for patients with cancers in a forest plot.

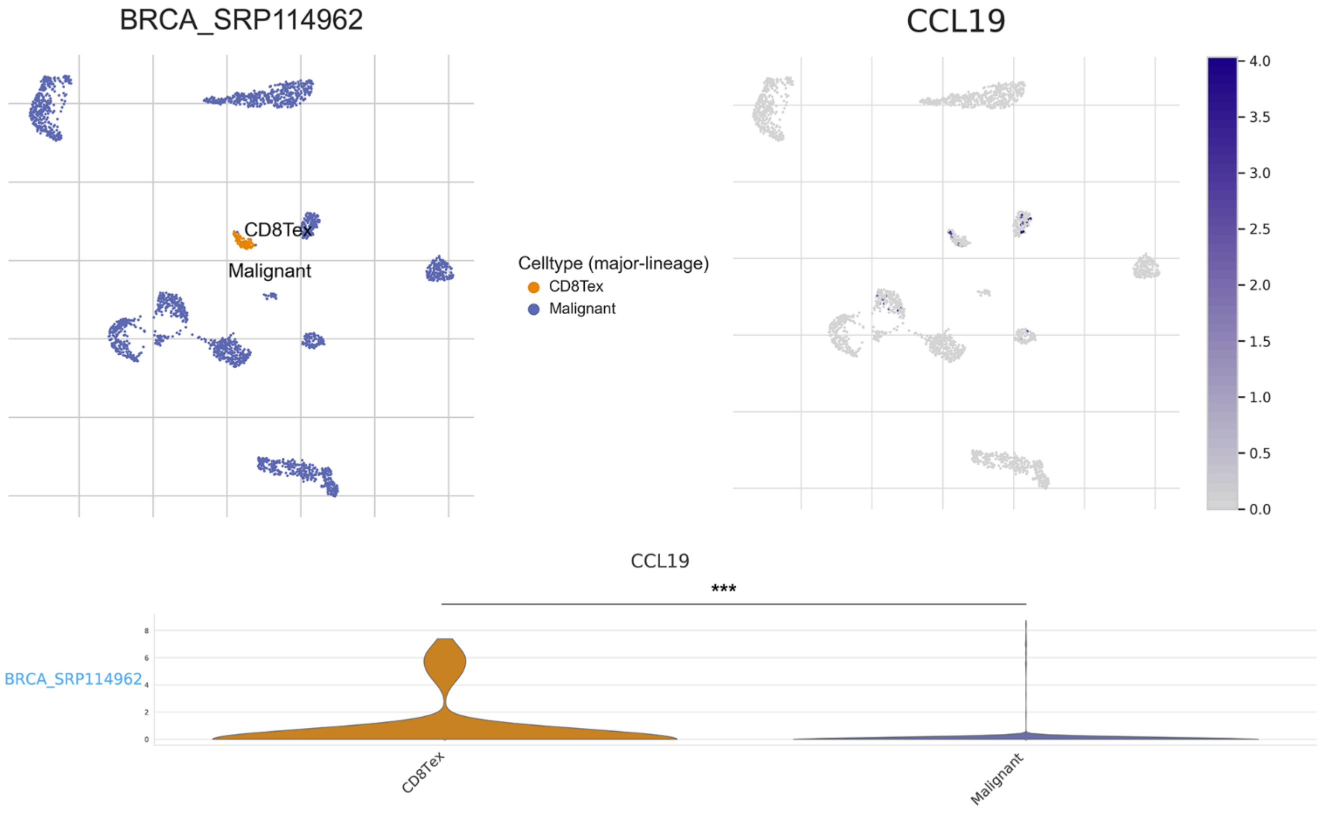

Supplementary Figure 3. Single-cell SRP114962 cohort was used to locate the CLL19 expression on CD8+ T cells. After identification of 2,472 cells from 8 BRCA patients, we found that CCL19 was mainly expressed in CD8+ T cells, and CD8+ T cell highly cover the tumor.
